# Unraveling Origins of EPR Spectrum in Graphene Oxide Quantum Dots

**DOI:** 10.3390/nano10040798

**Published:** 2020-04-21

**Authors:** Krzysztof Tadyszak, Andrzej Musiał, Adam Ostrowski, Jacek K. Wychowaniec

**Affiliations:** 1 Institute of Molecular Physics, Polish Academy of Sciences, ul. Smoluchowskiego 17, 60-179 Poznań, Poland; 2 School of Chemistry, University College Dublin, Belfield, Dublin 4, Ireland; jacek.wychowaniec@ucd.ie

**Keywords:** graphene oxide, quantum dots, EPR, magnetism, photoluminescence

## Abstract

Carbon nanostructures are utilized in a plethora of applications ranging from biomedicine to electronics. Particularly interesting are carbon nanostructured quantum dots that can be simultaneously used for bimodal therapies with both targeting and imaging capabilities. Here, magnetic and optical properties of graphene oxide quantum dots (GOQDs) prepared by the top-down technique from graphene oxide and obtained using the Hummers’ method were studied. Graphene oxide was ultra-sonicated, boiled in HNO_3_, ultra-centrifuged, and finally filtrated, reaching a mean flake size of ~30 nm with quantum dot properties. Flake size distributions were obtained from scanning electron microscopy (SEM) images after consecutive preparation steps. Energy-dispersive X-ray (EDX) confirmed that GOQDs were still oxidized after the fabrication procedure. Magnetic and photoluminescence measurements performed on the obtained GOQDs revealed their paramagnetic behavior and broad range optical photoluminescence around 500 nm, with magnetic moments of 2.41 µ_B_. Finally, electron paramagnetic resonance (EPR) was used to separate the unforeseen contributions and typically not taken into account metal contaminations, and radicals from carbon defects. This study contributes to a better understanding of magnetic properties of carbon nanostructures, which could in the future be used for the design of multimodal imaging agents.

## 1. Introduction

Quantum dots (QD) are nanoparticles (<100 nm), which have distinct electrical and optical properties to the parental materials with larger sizes, at which quantum mechanics is not playing a crucial role. One of the recognizable properties is the change of the photoluminescence frequency with the change in size of nanoparticles. This property is not only limited to the well-known semiconductor nanocrystals, e.g., PbS [1], CdSe [2], but was also observed in graphene nanoflakes and carbon nanodots [3,4,5]. Graphene-based materials due to their unique properties form their own branch of the QD family, and overcome many safety concerns in their unprecedented versatility of technological fields [6]. Applicability of graphene-based quantum dots can be increased by various oxidation protocols to form graphene oxide (GO) with a unique collection of oxygen rich groups on its surface (–COOH, –OH, =O, –O–) [7]. This material exhibits a vast number of properties including long time luminescence stability, ability to be well-dispersed in aqueous media, biocompatibility, and low toxicity [8,9], making it an important and promising material for biomedical applications [10]. Due to their properties, graphene oxide quantum dots (GOQDs) are excellent probes for high-contrast bioimaging and biosensing applications, [5,11,12] e.g., biosensors [13,14], and can be used in anticancer therapies [15,16]. GO allows obtaining a vast diversity of structures having specific/controllable biological properties [8,17]. Above that, graphene oxide synthesis is a straightforward process, taking place in an atmospheric pressure and at room temperature leading to possibilities of high-quality large-scale production.

From the methods of obtaining GOQDs two approaches can be distinguished: Firstly, the top-down approach, which means breaking larger flakes/particles usually by applying ultra-sonication with additional filtration (precursors can include: graphite [18], cellulose [19], or coal [20]). Secondly, the bottom-up approach by carbonization of carbon rich molecules or polymers, e.g., poly(3-alkylthiophenes) via oxidative polymerization [21], citric acid via microwave assisted synthesis [22], eco-friendly solvothermal process by autoclaving in ethylene glycol under 180 °C for 20 h [23], or microwave pyrolysis of poly(ethylene glycol) and saccharide (glucose, fructose) [24]. The possibility of tuning the optical, electrical, and hence opto-electrical properties permits the use of GOQDs for biological imaging, as opto-electrical single-photon detectors [25,26]. The optical properties of QDs are in the central field of study for multiple research groups, but in this article, we were interested in investigating the magnetic properties of QDs of graphene oxide obtained by a top-down technique and better understand the origins of magnetic signals. Previous experimental attempts included: Creating vacancies by irradiation [27,28], creating sp^3^ defects by surface doping with H and F ions [29,30], and adatoms such as C–OH groups [31,32,33,34]. Previously, we studied larger GO flakes interconnected into the form of aerogels [35], their spin relaxation processes [36,37], as well as other carbon materials, in which magnetic properties depended on particle size [38,39], and showed ferro- and anti-ferromagnetic properties [40,41].

## 2. Materials and Methods

### 2.1. Synthesis of GOQDs

GO water dispersion (4 mg/mL) was purchased from the NanoPoz Company (Poznań, Poland). Flake size distributions were obtained by manually measuring sizes using ImageJ^®^ from the sizes of around 200 flakes from multiple scanning electron microscopy images and the original flakes diameters were found in the range 0.8–46 µm, similar to studies using the same commercial flakes [17]. This dispersion was used to fabricate further GOQDs using the modified method described by Sun et al. [18]. Firstly, 40 mL of GO suspension was ultra-sonicated (Branson Digital Sonifier^®^ SFX 550, Warsaw, Poland) in an ice bath for 30 min (50% amplitude, 22 W). After 30 min, the ice batch was changed and the sonication repeated again for 30 min. Then, the dispersion was ultra-centrifuged (Thermo Scientific™ Sorvall LYNX 4000 Superspeed Centrifuge) at 24,000 rpm for 1 h. After that, the supernatant was subjected again to the 2 × 30 min sonication cycle with an ice bath change between the cycles, the dispersion was ultra-centrifuged one more time for 1 h, and the final supernatant was collected. Finally, 100 mL of HNO_3_ acid (Sigma-Aldrich, CAS Number: 7697-37-2) was added to the supernatant and the solution was kept at 160 °C (temperature of silicon oil bath) for 39 h, with the final 9 h dedicated to drying without a reflux condenser. The product of this process was brown powder, which was redispersed in 100 mL of water and then finally filtered through a 100 nm pore sized Minisart^®^ syringe filter. This solution was used as obtained for optical spectroscopy measurements, whereas for magnetic measurements a further step of drying at 70 °C for 12 h was used prior to measurements.

### 2.2. UV–Vis Spectroscopy

The absorption spectrum in the UV–Vis range of GOQDs water dispersion was recorded using a Lambda 950 UV/Vis/NIR spectrometer (ParkinElmer, Waltham, MA, USA) equipped with polystyrol/polystyrene cuvettes in the spectral range 200–1000 nm and 1 nm step. Cuvettes were purchased from SARSTEDTAG & Co. (Nümbrecht, Germany).

### 2.3. Fluorescence Spectroscopy

Fluorescence spectra were recorded with the Gilden Photonics FluoroSENS 9000 spectrometer (Glasgow, United Kingdom) equipped with a 150 W continuous Xenon Arc Lamp (Ozone Free, Osram XBO 150 W/CR OFR, (Munich, Germany) with 1 nm step in the range 400–850 nm.

### 2.4. Magnetization Measurements

Magnetic hysteresis loops and magnetization versus temperature were measured on 96 mg of brown GOQDs powder by a vibrating sample magnetometer in the Quantum Design Physical Property Measurement System (San Diego, CA, USA). Magnetic hysteresis loops were recorded in the field up to 2 T. Magnetization vs. temperature was measured in three magnetic fields 100, 300, and 500 Oe.

### 2.5. Scanning Electron Microscopy (SEM) and Energy Dispersive X-ray Spectroscopy (EDX)

Flake size distribution of GO/GOQDs was estimated using images obtained by the SEM Jeol 7001TTLS microscope (Akishima, Japan) working at a maximum of 30 kV. Energy dispersive X-ray spectroscopy (EDX) measurements were performed as follows: GO/GOQDs flakes were suspended on 200 μm thick pure gold substrate, forming a thick layer of GO/GOQDs flakes used only for this analysis. Measurements were carried out with an accelerating voltage of 20 kV, 2048 channels were used, detection was in the range 0–10 kV, and quant optimization was performed on silicon. A single EDX measurement was carried on a grid of 8 × 3 points (all 24 points were each accumulated during a 200 s collection time). For statistics, each measurement was performed five times and averaged. A gold background was subtracted from the final elemental composition. (Figure S1: SEM images of the graphene oxide flakes throughout the 3 steps of preparation of GOQDs.)

### 2.6. Electron Paramagnetic Resonance (EPR)

EPR spectra were recorded using the Bruker ELEXSYS E500 (Bremen, Germany) with helium cryostat ESR 900 (Abingdon, United Kingdom). Previously calibrated 2,2-diphenyl-1-picrylhydrazyl (DPPH) was used as a standard. Temperature was controlled using the ITC4 503S Oxford thermostat (Abingdon, United Kingdom). Spectra were always recorded with a modulation amplitude at least eight times lower than the line width with a microwave power low enough to avoid saturation. The last strongly over modulated spectrum was recorded at 5 K with 0.64 mW of microwave power and modulation amplitude set to 2 G to enhance the background signals. About 11 mg of dry brown powder were used.

## 3. Results and Discussion

### 3.1. GOQDs Fabrication

**Figure 1:**
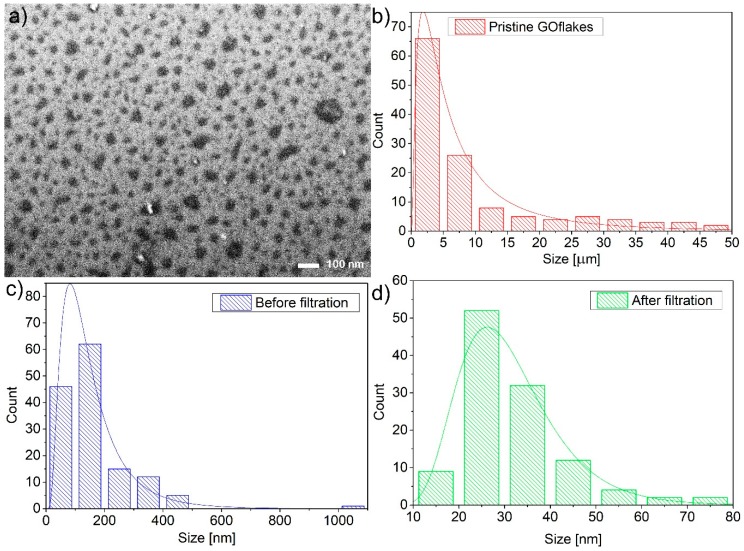
(**a**) SEM image of graphene oxide quantum dots (GOQDs) after filtration (100 nm cut-off) spread over doped silicon substrate. Flake size histogram of (**b**) GO flakes bought from Nanopoz (red dashed plot); (**c**) GO flakes after boiling in acid, ultra-centrifugation, and decantation; and (**d**) GOQDs after syringe filtration.

During all fabrication steps, i.e., (1) starting with the pristine GO sample; (2) before syringe filtration, but after ultra-centrifugation and decantation, and (3) after syringe filtration (with 100 nm cut-off), GO/GOQDs were drop-casted on a flat silica (SiO_2_) surface and imaged using the scanning electron microscopy (SEM) (Figure 1 and Figure S1). Subsequently, the statistical analysis of the flake sizes was performed and results were presented as histograms in Figure 1c,d and Table 1. The sizes of the pristine flakes obtained by the Hummers’ method (Nanopoz company) were in the range ~0.8–46 µm, with the mean value ~9.7 µm. A further process led to the decrease of flake sizes. After 39 h of HNO_3_ boiling the average size was reduced to ~157 nm, but still a small fraction had sizes exceeding ~1 µm. The narrowing of the final size distribution could only be done by a syringe filtration reaching maximum of the distribution (i.e., minimum size) at ~31 nm (length of 127 hexagonal units). The largest sized flakes did not exceed ~80 nm. It is clearly visible that each further filtration step decreases the ratio between the standard deviation (SD) and the mean flake size. The final filtration step narrowed the distribution the most and subsequently produced the GOQDs used in all further measurements.

Energy dispersive X-ray spectroscopy (EDX) revealed a similar elemental composition to GO provided by the same company [42]. The most dominant elements in GOQDs were 58% C; 40% O; 1% Na, and 1% S, the same within resolution limits of the technique to original GO flakes. It is worth noting that the residual presence of sulphur and sodium is expected due to the typical preparation route in the modified Hummers’ method using sulfuric acid and pH change washings with NaOH [43]. Other possible elements, such as manganese or iron, in lower quantities than < 1% cannot be reliably measured using this technique. For this measurement, a thicker GO layer was used on a gold support than shown in Figure 1a. Traces of remaining sodium and sulphur after the Hummers’ method were observed, however, no traces of manganese or iron were seen. Is it worth noting that previous EPR measurements on GO aerogels at 4.2 K revealed manganese-free samples [42].

### 3.2. Optical Measurements Confirm GOQDs

In the case of carbon nanodots, the source of PL is dominated by trap states in the bandgap and by the superposition of responses of assembled individual emitters (functionalization) and does not solely depend on HOMO-LUMO transitions [5]. Observed transitions can be modified by an external magnetic field, which is responsible for singlet-triplet mixing in GOQDs owing to the Zeeman splitting of triplet states [12].

**Figure 2:**
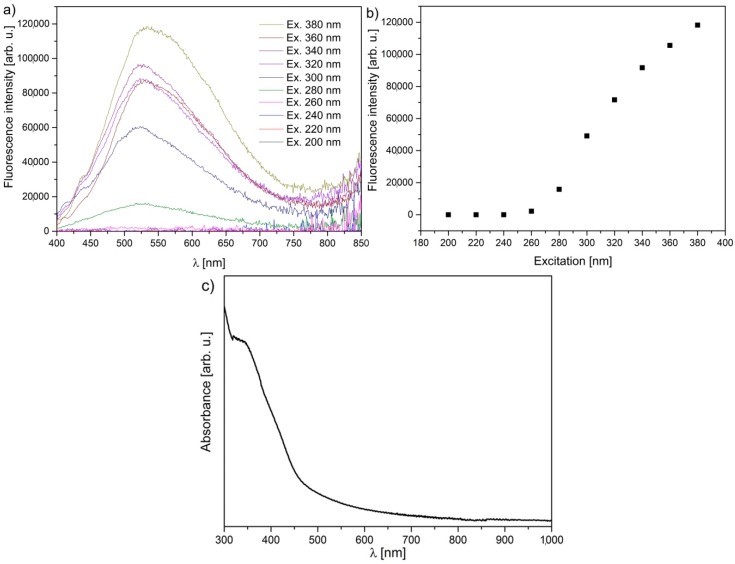
(**a**) Fluorescence plots for multiple excitation wavelengths; (**b**) maximum intensity as a function of the excitation wavelength; (**c**) optical absorbance spectrum in the UV–Vis range. All measurements were performed on obtained aqueous GOQDs.

The GOQDs photoluminescence (PL) spectrum can be decomposed to two-Gaussian, overlapping components [44] forming one broad spectrum with a maxima at around 500 nm stretched over 400 to 750 nm range (Figure 2a). The spectrum shape and properties are typically dependent on the level of thermal reduction [44,45] and hydrogen content [46]. If reduced at 180 °C for 3 h, the spectrum becomes narrower and shifts to shorter wavelengths with a maximum at 453 nm showing a quarter of the initial linewidth [45]. Chien et al. [44] demonstrated the change of the original yellow-red PL spectrum of GO to the blue spectrum of rGO, which was explained by a reduction in the number of disorder-induced defects in the π–π * gap and the change in sp^2^ to sp^3^ ratio upon reduction. Therefore, the GOQDs spectrum in this study consists of numerous disorder-induced defect states arising from original GO and processing techniques. The fluorescence intensity increases when the excitation wavelength gets closer to a fluorescence maximum, which further shifts from 505 (Ex. 260 nm) to 532 nm (Ex. 380 nm) (Figure 2b). The fluorescence FWHM is 193 nm in the 467–660 nm range. From the absorbance spectrum (Figure 2c), it is clear that GOQDs absorb in the 300 to 500 nm range with a long absorption shoulder reaching around 800 nm. The results suggest that the GOQDs are still oxidized (after 39 h of boiling in HNO_3_) compared to the other report, where samples were reduced at 180 °C for 3 h [45].

### 3.3. Magnetic Properties of GOQDs

**Figure 3:**
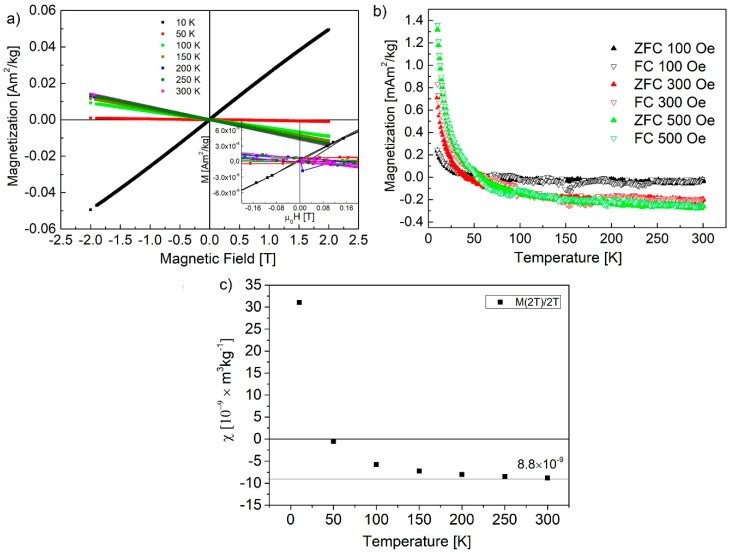
(**a**) Magnetization vs. magnetic field strength; (**b**) zero field cooling (ZFC) and field cooling (FC) dependences; (**c**) magnetic susceptibility recorded at 2 T for selected temperatures (*χ* = M (2 T)/2 T). All measurements were performed on obtained dried GOQDs.

Perfect graphene flakes are diamagnetic, which limits their applications in spintronic, however, reports show a more complicated magnetic behavior due to zigzag edges [37,47], which can give rise to the edge states magnetism and basal-plane sp^3^ defects formed by –OH groups (C–OH ~1–1.2 µ_B_) [36,48], and adatom-induced magnetism [31]. In comparison to large graphene and GO flakes, theoretical and experimental studies show that GOQDs are nonmagnetic, but the addition of defects and adatoms causes weak paramagnetism [18]. In our case, if the ferromagnetic behavior is observed due to the presence of a large number of –OH surface groups, then the proceeding reduction would only leave a weak paramagnetic signal (Figure 3). This is the case of moderately reduced samples [46], which can be observed here. The magnetic moment vs. magnetic field plot presented in Figure 3a shows a straight paramagnetic behavior with additional diamagnetic contribution at higher temperatures. Results presented by Wang et al. [46] show that it is easier to saturate the magnetization for GO than for the reduced GO, which is usually visible at around 6 T. The magnetic properties of GOQDs show mostly diamagnetic contribution coming from Langevin and Landau diamagnetism (Figure 3c). Magnetization plots recorded in the narrow field range (±0.2 T) show no hysteresis. Field cooling (FC) and zero field cooling (ZFC) curves do not show any temperature bifurcations, which means the magnetization vector is able to follow the magnetic field changes until the minimal measured temperature of 10 K. This indicates non-interacting, paramagnetic centers (Figure 3b). In the temperature range from 300 to 50 K, the main contribution of GOQDs is diamagnetic.

**Figure 4:**
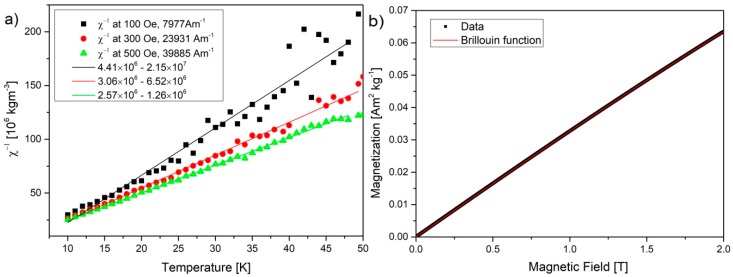
(**a**) Reversed magnetic susceptibility *χ*^−1^ as a function of temperature in the range 10–50 K measured at three different magnetic fields (indicated by different symbols); (**b**) magnetization as a function of magnetic field (0–2 T) fitted with the Brillouin function R^2^ = 0.9999. All measurements were performed on obtained dried GOQDs.

Figure 4a shows the plot of reciprocal magnetic susceptibility vs. temperature, obtained from data in Figure 3b. The straight line fit follows the data well and confirms paramagnetic behavior with a negative intercept, indicating additional weak magnetic exchange interactions.

Figure 4b shows the plot of the magnetization vs. H recorded at 10 K from which the diamagnetic contribution recorded at 300 K was subtracted. This data can be fitted with a Brillouin function:

$$M=M_{s}\left[\frac{2J+1}{2J}Coth\left(\frac{2J+1}{2J}x\right)-\frac{1}{2J}\left(\frac{x}{2J}\right)\right],$$

where: $x=\frac{gJ\mu_{B}H}{k_{B}T}$, $M_{s}=NgJ\mu_{B}$, g is the Landau factor, *J* is the angular momentum number, *N* is the number of spins, and *k_B_* is Boltzmann constant [18]. As seen in Figure 4b, a very good fit was achieved with the following parameters: *M_s_* = 0.22, *J* = 2.41, *N_s_* = 4.7 × 10^18^ spins/g, and *R*^2^ = 0.9999. The obtained saturation value equal to 0.22 Am^2^.kg^−1^ should be reached at around 6 T, as it was reported in the literature [18]. The *J* value of 2.41 higher than 0.5 is described in the literature [18], as a result of interactions between 4–5 spins (forming clusters). Similar values of *J* = 2, or with alternative fitting 5/2 were obtained by Panich et al. for nanographites doped with iron [49]. Further, it is stated that the increased annealing temperature > 200 K destroys large clusters and leads to a decreased value of J (0.5 at 1000 K) [45]. The assumption is that the thermal migration of defects and –OH groups through a basal plane disrupts clustering and formation of large magnetic moments.

The number of spins at 4.7 × 10^18^ spins/g is similar to the values obtained in the literature at 4.1–5.7 × 10^18^ [18] and 1.18–78.1 × 10^18^ spins/g (SQUID) and 1.1–3.7 × 10^18^ (EPR) for nanographites doped with iron [49]. The number of radicals belonging to the sp^3^ defects (central EPR line 2.0045) on the surface that was measured in here with EPR, was 8.05 ± 1.2 × 10^14^ spins/g (±15% inaccuracy [49,50]). The difference was 4.6972 × 10^18^ spins/g and it belongs to the iron and manganese ions present in the dried dispersion. The presence of the metal ions in the sample is justified by taking into consideration all the preparative steps, which damaged larger flakes, opened the pores in between double and triple stacked flakes, released contaminations in the solution, and further in the process removed larger flakes leaving only contaminated dried small flakes. In this regard, EPR is an indispensable tool allowing the distinction of signals coming purely from GOQDs defects. The measured J value as 2.41 is close to the J value of high spin iron Fe^3+^ S = 5/2 (it can exist also as 3/2 and Fe^2+^ with S = 2 and 0), as well as Mn^2+^ = 5/2. The total number of spins detected in magnetic susceptibility measurements is the same as that reported in the literature. We address differently the source of the signal and separate the contributions. Small intercepts visible in Figure 4a and lack of differences in ZFC and FC suggest that metals exist in the form of single ion complexes. From the EPR measurement, it is known that only one of 5.8 × 10^3^ spins belong to the radical system in which we are interested. Statistically, one magnetic defect can be found in 2112 flakes or 14,705 hexagons or 29,410 carbon atoms (taking 31.2 nm as flake diameter).

The total number of spins, which are made of radicals and metal ions, is estimated at 4.8 ± 0.7 × 10^18^ spins/g (EPR) and fits well with the susceptibility measurements. Due to the fact that EPR can easily separate different paramagnetic contributions having different g-factors, a further analysis is made only for the radical line. The EPR signal stemming from sp^3^ defects is located at a g-factor of 2.0045 at room temperature. A slightly higher g-factor than for free electrons (2.0023) is indicative of small positive spin-orbit coupling λ typical for organic radical species. The g-factor decreases insignificantly to 2.0037 (5 K) with the temperature decrease. The number of spins corresponding to the central radical (8.05 ± 1.2 × 10^14^ spins/g) is lower than that for fresh pristine graphene flakes 4 × 10^20^ spins/g [48], but larger than the value 1.5 × 10^13^ spins/g estimated for relaxed/aged graphene flakes reported in the literature [37].

**Figure 5:**
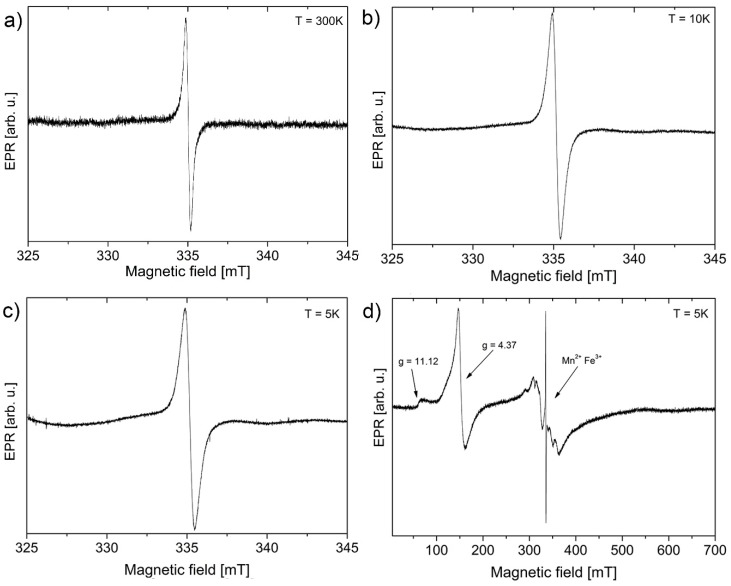
Electron paramagnetic resonance (EPR) lines recorded at (**a**) 300, (**b**) 10, (**c**) 5, and (**d**) 5 K over the full range with maximal gain. All measurements were performed on obtained dried GOQDs.

The EPR line has a Lorentzian shape in the entire temperature range (Figure 5a–c). The source of the signal is assigned to moments on sp^3^ defects, as mentioned previously, to the conduction of electrons. The second source, which for bulk, conductive samples leads to an asymmetric Dyson line shape [51], does not appear here due to lack of magnetic field gradient over electrically separated GO flakes (separated low conductive flakes) [52]. A similar statement can be found in the article written by Shames et al. [53] for 40 nm multi-shell onion structures, where one of the Lorentzian lines was assumed to belong to conduction electrons [53]. Therefore, according to this theory, the conduction electrons in this case would also exhibit a Lorentzian line shape [38].

Figure 5d shows metal contamination, which appears clearly in the lowest temperature measured at 5 K but is also visible at 300 K with a strong amplification and large modulation amplitude used only for the magnification of the background signals. The additional signals were recognized as coming from Fe^3+^ ions with an effective g-factor of ~4.37 and low (rhombic) site symmetry while the signal at ~11.12 can originate from the same ions in axial site symmetry [54,55]. It was stated in the literature by Panich et al. [49] that nanographites (<30 nm) can anchor (6.6 times) more iron ions than larger micrometre sized particles on the edges.

The broad multiline signal at g-factor ~2 is characteristic for Mn^2+^ ions [56,57,58,59,60]. It was reported by Panich et al. [59] that Mn^2+^ ions anchor onto the graphene surface forming charge transfer complexes, which further contribute to the ^13^C relaxation time. Mn^2+^ ions do not form agglomerates and rather exist in ion form even in a higher concentration than measured here [59].

Iron is the most basic contamination of carbon and could exist in the sample from the beginning trapped between multilayer GO flakes (or between original graphite), while manganese was added during preparation of graphene oxide by the Hummers’ method. Previous studies performed on GO aerogels obtained from the same GO [35,36,42] did not show any metal contaminations even at 4.2 K and at highest magnifications. Further, the contamination source could be the large power ultra-sonication tip, in which GO suspension was ultra-sonicated. For clarity, such small metal contamination levels cannot be detected by EDX due to the enormous ratio of compounds of interest to contamination. As mentioned previously, lack of hysteresis loop allows the estimation of the metal contamination in the form of ion complexes and not (nano)particles. No other effects such as ferro- and anti-ferromagnetic ordering as well as differences in FC and ZFC electron spins, which were reported in the literature in graphene [47,61] and other natural carbons [41], were observed here.

**Figure 6:**
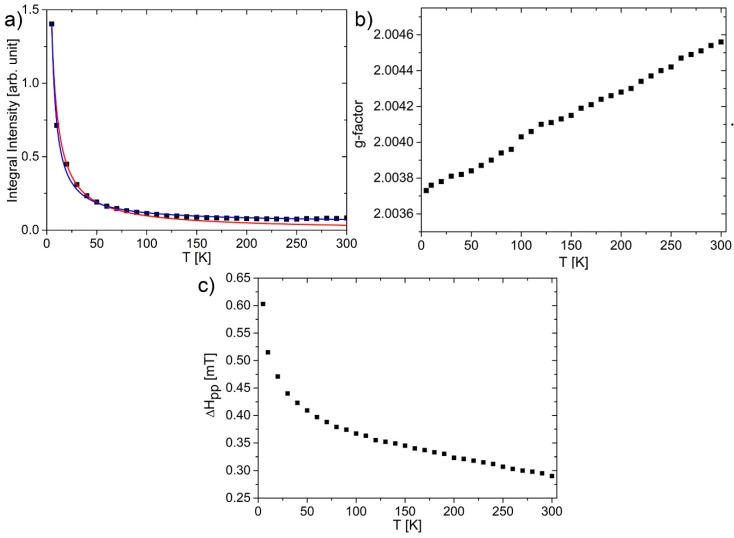
(**a**) Temperature dependence of integral intensity. Curie–Weiss relation (red), Sum of Pauli, and Curie contributions (blue); (**b**) g-factor of the central GOQDs line; (**c**) peak–peak linewidth. All measurements were performed on obtained dried GOQDs.

The integral intensity was estimated using the relation: $I=Amp\times \Delta H_{pp}^{2}$(Figure 6). The integral intensity can be fitted using two contributions: Curie and temperature independent Pauli contribution: $I=\frac{C}{T}+B$, where *C* = 6.8 and *B* = 0.05 give the R^2^ parameter 0.997 (Figure 6). If comparing the intensities of both contributions the results were as follows: At 5 K, localized centers (Curie contribution) to delocalized centers (Pauli contribution) is 1.36/0.05 = 27.2 times larger in favor for localized centers, although at 300 K it is already 0.022/0.05 = 0.45. A large source of the signal at room temperature originates from conduction electrons. For comparison, Curie–Weiss functions in the form: $I=\frac{C}{T-\theta }$, where *C* = 10 and *θ* = 2.3*K* give a slightly lower R^2^ value of 0.984.

The signal width was increasing with the temperature decrease, which is connected to the increase of relaxation T_2_^−1*^ rate. In this case, by applying the Bloch equation [37], the relaxation rate is changing from 0.7 kHz at room temperature to 1.45 kHz at helium temperature.

Comparing the results to other graphene-based systems, it can be seen that the relaxation for GOQDs is much slower. Panich et al. [59] reported that graphene oxide (flake size undefined) heavily doped with Mn^2+^ ions as an “anti-saturation” behavior, which suggests a very short electron relaxation time T_1_ (from saturation measurements: <10 ns, $T_{1}^{-1}$ = 100 MHz, for nanographites doped with Fe^2+,3+^ T_1_ < 10^−9^ s [49]). Further, graphene nanoribbons exhibit similar, but two spin-lattice relaxation processes: First with the rate T_1_^−1^ ~0.014 MHz and a second component with the relaxation rate 0.1 MHz [62], graphene T_1_^−1^~T_2_^−1*^~3 MHz [37], both at 100 K. In partially reduced graphene oxide (prGO) with flakes reaching 46 µm (average ~2 µm) this rate is 17.5 MHz (T_1_^−1^ = T_2_^−1^) [35]. Reduction causes the increase of the spin-lattice relaxation rate T_1_^−1^ by a factor of ~5.8 [35], due to the larger number of surface defects/holes, and inhomogeneity caused by the remaining oxygen and hydrogen-rich groups. By careful sample preparation, extremely long relaxation times were reported for graphene oxide paper [36]. The spin-lattice relaxation time is found to decrease from 52 ms at 5 K to 0.153 ms at 240 K and is dominated by the direct process below 100 K. For comparison with previous values at 100 K, the T_1_^−1^ was 535 Hz [36]. A general statement from the discussion is that the reduction process increases the number of defects and further increases the relaxation rate, but decreasing the flake size removes paramagnetic defects from the surface and edges decreasing the relaxation rate and overall paramagnetic behavior, leaving mostly a diamagnetic response to magnetic field in GOQDs.

## 4. Conclusions

In summary, we showed that the preparation procedure of graphene oxide quantum dots from parental graphene oxide did not change the C/O ratio (EDX). GOQDs dispersion exhibited a strong fluorescence at ~500 nm over excitation wavelengths of 280–380 nm. The dominant magnetic response of GOQDs (vibrational magnetometer) was diamagnetic in the temperature with 300–50 K range and below 50 K of dominated paramagnetic contribution. Diamagnetism comes due to the Langevin and Landau diamagnetism, and paramagnetic contribution originates from the Curie and Pauli paramagnetism. The magnetic susceptibility (EPR integral intensity) increased with the temperature drop and was weaker than the Curie function predicted, which can be explained by the influence of conduction electrons contribution to magnetic susceptibility. The radical signal at g = 2 can be separated from additional metal contamination in EPR spectroscopy. A high J value of 2.41 is explained by the major metal ion contribution to magnetic susceptibility measurements and not by formation of clusters by radical spins. These results indicate that GOQDs would require chemical treatment and/or metal doping for their subsequent use as bimodal bio-imaging agents. Finally, we show that EPR is a necessary tool for studying magnetic properties of carbon nanostructures due to its intrinsic capability of distinguishing magnetic signals from defects, compared to unforeseen contaminations, and that it is necessary to check the state of the sample using this technique to understand the origins of magnetic signals.

## Figures and Tables

**Table 1:** GO and GOQDs flake size statistic.

Sample	Maximum of Lognormal Distribution [nm]	Mean [nm]	SD [nm]	SD/Mean [%]	Minimum [nm]	Median [nm]	Maximum [nm]
**Pristine GO**	1822	9771	11,363	116	709	4613	46,366
**GO after boiling in acid, ultracentrifugation, and decantation**	82	157	125	79	33	128	1047
**GOQDs**	26	31	12	38	13	29	77

## Supplementary Materials

The following are available online at https://www.mdpi.com/2079-4991/10/4/798/s1, Figure S1: SEM images of the graphene oxide flakes throughout the 3 steps of preparation of GOQDs.
