# Supplementary material for: Unraveling Origins of EPR Spectrum in Graphene Oxide Quantum Dots
Source: Nanomaterials (Basel). 2020 Apr 21;10(4):798. doi: 10.3390/nano10040798 (PMC7221827; doi:10.3390/nano10040798)
Supplement: Supplementary file 1 [file nanomaterials-10-00798-s001.pdf]

nanomaterials

supplymentary

Unraveling Origins of EPR Spectrum in Graphene Oxide Quantum Dots

Krzysztof Tadyszak [1,*], Andrzej Musiał [1], Adam Ostrowski [1] and Jacek K. Wychowaniec [2]

[1] Institute of Molecular Physics, Polish Academy of Sciences, ul. Smoluchowskiego 17, 60-179 Poznań, Poland; andrzej.musial@ifmpan.poznan.pl (A.M.); adam.ostrowski@ifmpan.poznan.pl (A.O.)

[2] School of Chemistry, University College Dublin, Belfield, Dublin 4, Ireland; jacek.wychowaniec@ucd.ie

* Correspondence: krzysztof.tadyszak@ifmpan.poznan.pl

Received: 19 February 2020; Accepted: 17 April 2020; Published: 21 April 2020

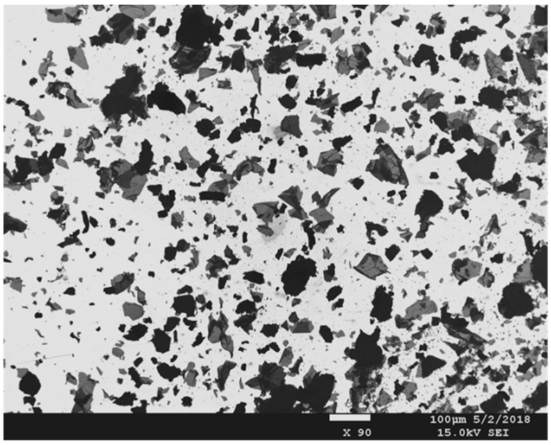

1) pristine GO sample

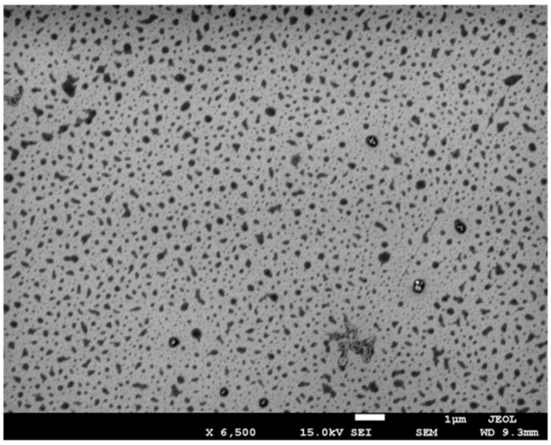

2) before syringe filtration, but after ultra-centrifugation and decantation

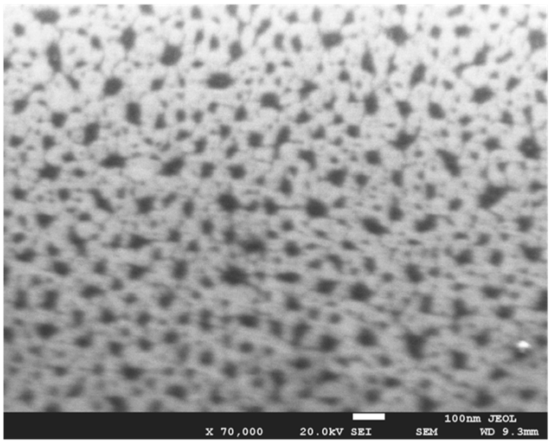

3) after syringe filtration (with 100 nm cut-off)

Figure S1. SEM images of the graphene oxide flakes throughout the 3 steps of preparation of GOQDs.
